# Sleep patterns correlates with the efficacy of tDCS on post-stroke patients with prolonged disorders of consciousness

**DOI:** 10.1186/s12967-022-03710-2

**Published:** 2022-12-15

**Authors:** Jie Yu, Yuehao Wu, Biwen Wu, Chuan Xu, Jiaye Cai, Xinrui Wen, Fanxia Meng, Li Zhang, Fangping He, Lirong Hong, Jian Gao, Jingqi Li, Jintai Yu, Benyan Luo

**Affiliations:** 1 Department of Neurology, First Affiliated Hospital, Zhejiang University School of Medicine, Hangzhou, 310003 Zhejiang China; 2 Department of Neurology, First People’s Hospital of Linping District, Hangzhou, 310003 Zhejiang China; 3 Center for Sleep Medicine, Sir Run Run Shaw Hospital, Zhejiang University School of Medicine, Hangzhou, 310003 China; 4 Rehabilitation Medicine Center, Department of Rehabilitation Medicine, Zhejiang Provincial People’s Hospital, Affiliated People’s Hospital of Hangzhou Medical College, Hangzhou, Zhejiang China; 5 Department of Rehabilitation, Hangzhou Hospital of Zhejiang Armed Police Corps, Hangzhou, 310051 China; 6 Department of Rehabilitation, Hangzhou Mingzhou Brain Rehabilitation Hospital, Hangzhou, 311215 China; 7 Department of Neurology and Institute of Neurology, Huashan Hospital, Shanghai Medical College, Fudan University, Shanghai, 200031 China

**Keywords:** Stroke, Sleep, Disorders of consciousness, tDCS, Prognosis

## Abstract

**Background:**

The subclassification of prolonged disorders of consciousness (DoC) based on sleep patterns is important for the evaluation and treatment of the disease. This study evaluates the correlation between polysomnographic patterns and the efficacy of transcranial direct current stimulation (tDCS) in patients with prolonged DoC due to stroke.

**Methods:**

In total, 33 patients in the vegetative state (VS) with sleep cycles or without sleep cycles were randomly assigned to either active or sham tDCS groups. Polysomnography was used to monitor sleep changes before and after intervention. Additionally, clinical scale scores and electroencephalogram (EEG) analysis were performed before and after intervention to evaluate the efficacy of tDCS on the patients subclassified according to their sleep patterns.

**Results:**

The results suggest that tDCS improved the sleep structure, significantly prolonged total sleep time (TST) (95%CI: 14.387–283.527, *P* = 0.013) and NREM sleep stage 2 (95%CI: 3.157–246.165, *P* = 0.040) of the VS patients with sleep cycles. It also significantly enhanced brain function of patients with sleep cycles, which were reflected by the increased clinical scores (95%CI: 0.340–3.440, *P* < 0.001), the EEG powers and functional connectivity in the brain and the 6-month prognosis. Moreover, the changes in NREM sleep stage 2 had a significant positive correlation with each index of the β band.

**Conclusion:**

This study reveals the importance of sleep patterns in the prognosis and treatment of prolonged DoC and provides new evidence for the efficacy of tDCS in post-stroke patients with VS patients subclassified by sleep pattern.

*Trial registration* URL: https://www.clinicaltrials.gov. Unique identifier: NCT03809936. Registered 18 January 2019

**Supplementary Information:**

The online version contains supplementary material available at 10.1186/s12967-022-03710-2.

## Background

Patients who suffer from severe brain damage may develop disorders of consciousness (DoC), including the vegetative state (VS) and the minimally conscious state (MCS) [1]. This state is defined as prolonged DoC if it lasts more than 28 days. Patients in VS retain behavioral sleep–wake cycle but are unaware of themselves or their environment [2], whereas those in MCS have reproducible signs of awareness and exhibit fluctuations in consciousness [3]. Stroke, especially cerebral hemorrhage, resulted in a worse prognosis for patients with prolonged DoC [4] compared to traumatic brain injury. Unfortunately, there is no effective treatment to improve the brain function of post-stroke patients in prolonged DoC.

Neuromodulation techniques such as repetitive transcranial magnetic stimulation (rTMS), transcranial direct current stimulation (tDCS) and deep brain stimulation (DBS) have been widely used in the treatment of prolonged DoC [5–7]. Of these, tDCS induces an excitatory stimulation of the cortical region of interest, and is non-invasive, easily operated and suitable for bedside treatment [8]. However, an investigation of its curative effect on prolonged DoC patients in previous studies contained many confounding factors, such as the cause, site of injury and some physiological conditions [9]. These biases may contribute to the uncertainty of the results, and few studies are based on subclassification.

Different from the physiological state, many DoC patients, especially VS patients, usually have no complete sleep cycles [10]. Some of them sustained wakefulness as found on electroencephalogram (EEG), even if they exhibited eye opening and closure [11]. Previous studies reported that more organized sleep patterns in prolonged DoC, especially NREM sleep stage 2, might predict positive outcomes [12, 13]. In addition, the brain regions associated with consciousness, such as the frontal lobe, thalamus, dorsolateral pons, are also involved in sleep regulation [14]. It suggested that the integrity of the brain network during sleep may reflect the plasticity of consciousness-supporting brain networks. Therefore, a subclassified cohort study based on sleep patterns was carried out.

To address this knowledge gap, we first monitored the overnight sleep of post-stroke patients in VS by use of polysomnography (PSG) and divided them into two groups according to the presence of sleep cycle. Then, we compared the recovery of their clinical scale scores, sleep patterns, and EEG power, functional connectivity before and after intervention, exploring the efficacy of tDCS on prolonged DoC subclassified by the sleep patterns. We hypothesized that the VS patients with sleep cycles have better response to tDCS than those without sleep cycles, which may be reflected by better clinical scores, EEG powers, functional connectivity and 6-month prognosis.

## Methods

### Study design and participants

This exercise study is a prospective, randomized, controlled trial (NCT03809936). Written informed consent was obtained from each patient’s legal guardian. This study was approved by the Ethics Committee of the First Affiliated Hospital, School of Medicine, Zhejiang University, Hangzhou Hospital of Zhejiang Armed Police Corps, China, and Hangzhou Mingzhou Brain Rehabilitation Hospital.

Patients were included according to the following criteria: age between 18 to 75 years old; maintained DoC state more than 1 month prior to enrollment; admitted to the hospital at least one weeks to eliminate interference from the new environment for sleep. Exclusion criteria were as follows: patients with unstable consciousness state (There are signs of spontaneous recovery or deterioration within 1 week); epileptic occurred within a week; people with the history of sleep disorders, schizophrenia, schizoaffective disorder or primary affective disorder; combined severe heart, brain, liver, kidney and hematopoietic system diseases or other serious primary diseases; patients who received sedatives such as nitrazepam, and some special treatment, such as transcranial magnetic stimulation (TMS) and transcranial direct current stimulation (tDCS).

All patients were recruited from the rehabilitation units of Hangzhou Hospital of Zhejiang Armed Police Corps**,** China, and Hangzhou Mingzhou Brain Rehabilitation Hospital in the period from April 2019 to December 2020 who fulfilled the above criteria were initially enrolled in the study. Glasgow Coma Scale (GCS) was used for assessment of the severity of brain injury in acute stage and Glasgow Outcome Scale-Expended (GOS-E) was used for assessing their prognosis [15]. Diagnosis of VS and MCS based on the five assessments within 10 days by DoC experts using the Coma Recovery Scale-Revised (CRS-R) by two trained and experienced neurologists [3]. Finally, 98 DoC patients were conducted, which contained 70 VS patients and 28 MCS patients (Additional file 3: Figure S1). The demographic and clinical characteristics which were from our database of every subject were listed in Additional file 1.

### Randomization and procedures

After obtaining written consent, patients were enrolled in the study and randomized using a computer-generated list into either the tDCS groups or the sham groups. Physicians and nurses were aware of the group assignment. Principal investigators enrolled participants and assigned participants to intervention. Assessment professionals and data analysis researchers were blinded.

### Intervention: tDCS and sham stimulation

All patients were divided into two categories based on their sleep cycles: the patients with sleep cycles and without sleep cycles. As our previous study [5], tDCS was administered using a DC-stimulator (neuroConn GmbH, Ilmenau, Germany). For real tDCS groups, direct current was applied using a battery-driven constant-current stimulator through saline-soaked surface sponge electrodes (7 cm × 5 cm) with the anode placed over the left dorsolateral prefrontal cortex (dlPFC) (F3 according to the 10–20 international EEG system) and the reference cathode placed over the right supraorbital region (Fp2) [16]. The current was increased to 2 mA at the onset of stimulation for 20 min per session [5]. For the sham tDCS groups, the same stimulation parameters were employed, except that the stimulator had a built-in placebo mode; when it was activated, two ramp fade-in/fade-out periods at the beginning and the end of sham stimulation mimicked the somatosensory artifact of real tDCS [17]. Both the real tDCS groups and sham groups were administered once a day, for 10 days.

### Sleep monitoring and interpretation

A continuous polysomnographic recording included eight channels of EEG electrodes (F3, F4, C3, C4, O1, O2, and both mastoids, which also are the reference and the ground electrodes); two channels of electrooculography (EOG), with the electrodes positioned 1 cm lateral and below and above to the outer canthi of both eyes; three channels of electromyography (EMG) with the electrodes placed on the chin and other two channels on the legs for periodic leg movement. Electrocardiography (ECG) and thorax and abdomen respiratory belts were used to monitor vital signs during sleep recording. To avoid the disturbance of sleep caused by rehabilitation treatment during the day, nighttime sleep was recorded in our study. According to the light-out time of the hospital and some nursing treatments in the morning, a total of  10 h sleep from 8:00 p.m. to 6:00 a.m. was used for analysis finally. The data were recorded using a mobile polysomnography device (NOX A1, NOX medical, Iceland) with a 256 Hz sampling rate. All recordings were performed in a separate, quiet room. The experimenter checked electrode impedance less than 5 kΩ.

The PSG traces in our recording were scored in 30-s epochs by two expert clinical neurophysiologists who have been engaged in sleep interpretation work and have rich experience in the interpretation of EEG in patients with severe brain injury. They were blinded to the patient’s clinical assessments. Following the AASM rules as much as possible [18], the two raters independently staged each recording and the traces were subsequently revised, if there was disagreement between the staging of epochs and the recognition of arousals. Uncertain epochs were not considered in the statistical analyses. Given to the particular nature of the sleep patterns in DoC patients, some adjustments were conducted. More specifically, we defined that the patients have no sleep cycle in 24 h if only wake stage or SWS patterns were presented within the range of our records. In addition, we defined that the patients have sleep cycles in 24 h if they have more than two transformations of sleep stages. Finally, total sleep time (TST), the duration of the REM, NREM2 and SWS stages of sleep, and of the number of phase transitions and arousals and other sleep parameters were quantified.

### EEG data recordings and analysis

EEG signals were recorded using a 64-electrodes BrainCap (Brain Products DmbH, Munich, Germany) in the international 10–20 system, and 1 of the 64 electrodes was placed under the right eye to record electrooculogram (EOG). Electrode impedances were kept below 10 kΩ. Preprocessing EEG data refers to our previous research [19].

EEG power spectrum density (PSD) were generated using the Welch’s method (one of the Fourier transforms) [20]. The different frequency bands on the frequency spectrum were divided by 0.1–46 Hz to obtain the relative powers. Power values were then averaged across each frequency band: delta (1–4 Hz), theta (4–8 Hz), alpha (8–12 Hz) beta (12–30 Hz) and gamma (31–45 Hz) and were averaged across all epochs.

Functional connectivity assesses functional communication between brain regions by estimating the level of synchronization of the EEG signals [21].

Coherence (Coh): For two different signals A and B, the coherence at each frequency (f) is defined as

$$Coh (f) = \mid SAB (f){\mid }^{2}= (SAA (f)SBB (f))$$

$\mid$ *S*_*AB*_* (f)*$\mid$ is the cross-spectral density between signals A and B; *S*_*AA*_ is the autocorrelation of signal A; *S*_*BB*_ is the autocorrelation of signal B [22].

Phase locking value (PLV) describes the distribution or variability of the relative phase [5] according to

$$PLVxy = \mid <e i\Delta \varphi r \left(t\right)>\mid = \mid \frac{1}{N}{\sum }_{n=1}^{N}{e}^{i\Delta {\varphi }_{r} ( {t}_{n} )} \mid$$

All of the analysis were conducted in the MATLAB software (The MathWorks, Natick, MA).

### Outcomes

The primary outcome was completion of the tDCS treatment and post-treatment assessments including CRS-R scores, sleep monitoring, and resting-state EEG.

Functional outcome was assessed 6 months after discharge using the Glasgow Outcome Scale Extended (GOS-E). The participants were usually seen in the rehabilitation unit by a professional doctor unaware of group assignment and otherwise they were contacted by telephone.

### Statistical analysis

Continuous variables were reported as means ± standard deviation (SD), and demographic characteristics were made using one-way analysis of variance (ANOVA) or Chi-square tests. Analysis of covariance (ANCOVA) was used for statistical comparisons and age was included as a covariate. For correlation analysis, Pearson correlations was performed. Statistical analysis was performed using SPSS version 23.0. *P* values of less than 0.05 after multiple-comparison correction using the false discovery rate method were considered significant.

## Results

In this study, the sleep patterns of 70 VS and 28 MCS patients after stroke were recorded overnight. All the MCS patients and 39 of the VS patients had sleep cycles within 24 h even though their sleep patterns were abnormal. In contrast, 31 VS patients had no distinct sleep cycles throughout the night, even though some of them had behavioral eye closure during the recording (Additional file 1).

### Cohort description

**Fig. 1:**
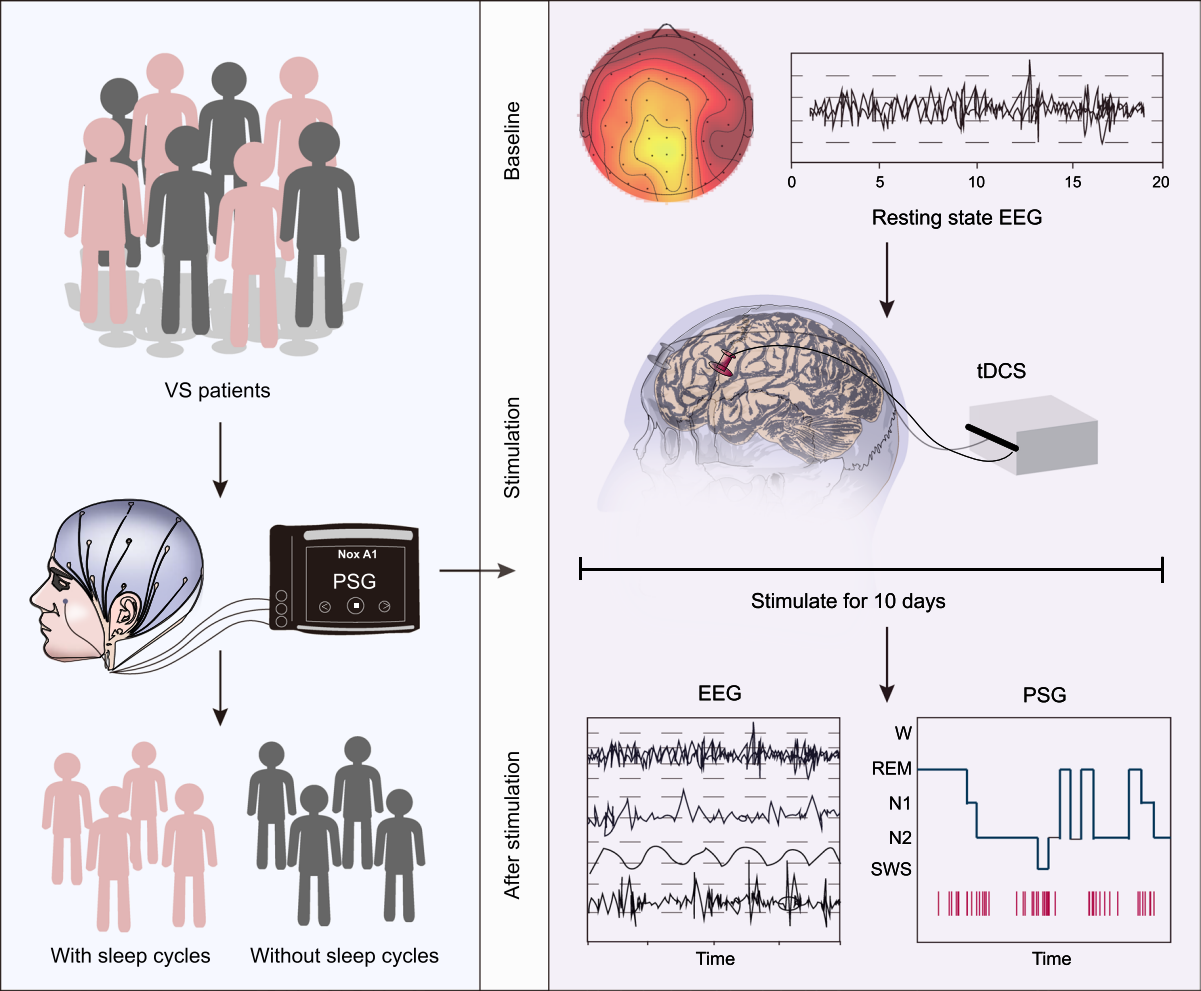
Flowchart of tDCS for VS patients subclassified by sleep pattern in this work. tDCS: transcranial direct current stimulation; PSG: polysomnography; VS: vegetative state

Numerous previous studies have shown that sleep deprivation is detrimental to brain recovery in patients who suffered from stroke [23, 24] and it also influences the efficacy of tDCS on patients [25, 26]. To investigate this concept, a total of 33 VS patients were divided into four subgroups according to their sleep patterns: VS patients with sleep cycles (Example in Additional file 3: Figure S1A), VS patients without sleep cycles (Example in Additional file 3: Figure S1B) and the corresponding sham groups. Their resting-state EEG data at baseline were simultaneously collected. After a 10-day tDCS or sham stimulation, the CRS-R scores, sleep patterns, and resting-state EEG data of each group were collected again to investigate the efficacy of the treatment (Fig. 1). The clinical characteristics of all VS patients in these groups are shown in Table 1. There was no significant difference in gender, age, GCS, CRS-R scores, and duration of DoC between sham and tDCS groups.

**Table 1:** Clinical characteristics of VS patients in sham groups and tDCS groups

	Sham groups		tDCS groups		*P* value
	Have sleep cycles	No sleep cycles	Have sleep cycles	No sleep cycles	
Patients(n)	10	6	10	7	/
Male (n, %)	8 (80.0%)	5 (83.3%)	10 (100.0%)	7 (100.0%)	0.313
Age (years)	58.4 ± 10.2	61.0 ± 8.7	52.4 ± 10.3	47.8 ± 8.5	0.065
GCS	6.6 ± 1.4	7.1 ± 0.8	6.7 ± 1.3	6.6 ± 1.3	0.421
CRS-R	5.3 ± 1.6	5.8 ± 0.8	5.5 ± 1.4	6.2 ± 1.3	0.495
Duration	159.8 ± 96.4	220.7 ± 95.5	188.2 ± 187.1	190.4 ± 128.3	0.858
Cause	Stroke	Stroke	Stroke	Stroke	/

Continuous variables are expressed as the mean ± standard error of mean (SD); *P* < 0.05 was considered statistically significant. tDCS: Transcranial Direct Current Stimulation; GCS: Glasgow Coma Scale; CRS-R: Coma Recovery Scale-Revised scores; GOS-E: Glasgow Outcome Scale-Extended; VS: vegetative state; MCS: minimally conscious state

### Clinical outcomes

**Fig. 2:**
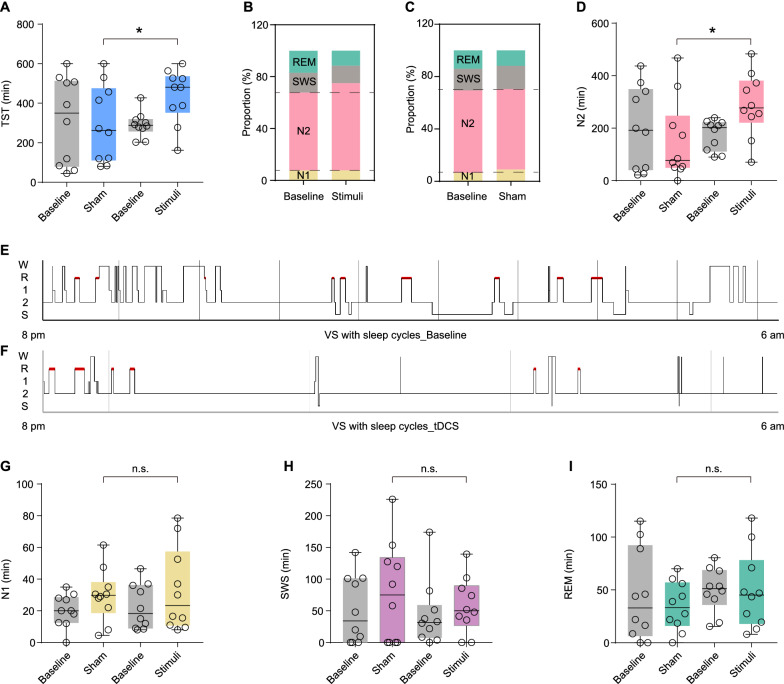
Changes in sleep pattern of VS patients with sleep cycles before and after treatments. **A** Total sleep time of VS patients with sleep cycles before and after treatments. Sleep structure of VS patients with sleep cycles in tDCS group (**B**) and sham group (**C**). **D** NREM sleep 2 stage of VS patients with sleep cycles before and after treatments. **E** Sleep pattern of VS patients at baseline in tDCS group. **F** Sleep pattern of VS patients after tDCS. Other Sleep stages of VS patients with sleep cycles before and after treatments, including NREM sleep 1 stage (**G**), slow-wave sleep (**H**), REM sleep stage (**I**). *P < 0.05, n.s: no significant difference; *P* values were determined using analysis of covariance (ANCOVA) and age was included as a covariate; TST: total sleep time; W: wake stage; N1: nonrapid eye movement sleep 1 stage; N2: nonrapid eye movement sleep 2 stage; SWS: slow-wave sleep; REM: rapid eye movement sleep; tDCS: transcranial direct current stimulation; VS: vegetative state

When comparing the sleep patterns of the patients at baseline and after treatments, no changes were observed in the VS patients without sleep cycles, whether they were in the sham group or the tDCS group (Additional file 2). The results suggested that the sleep structure of these patients could not be remodeled by tDCS. Interestingly, for the VS patients with sleep cycles, the total sleep time (TST) was increased after a 10-day tDCS treatment (95%CI: 14.387–283.527, *P* = 0.005, Fig. 2A), and the sleep structure significantly changed in tDCS group (Fig. 2B) compared to the sham group (Fig. 2C). In addition, for patients with sleep cycles in tDCS group, the NREM stage 2 was prolonged after a 10-day tDCS (95%CI: 3.157–246.165, *P* = 0.040, Fig. 2D). For example, the sleep patterns before and after tDCS of one patient in this group were shown in Fig. 2E, F. However, these changes were not observed in the other sleep stages, such as NREM stage 1 (Fig. 2G), SWS (Fig. 2H) and REM stage (Fig. 2I).

**Fig. 3:**
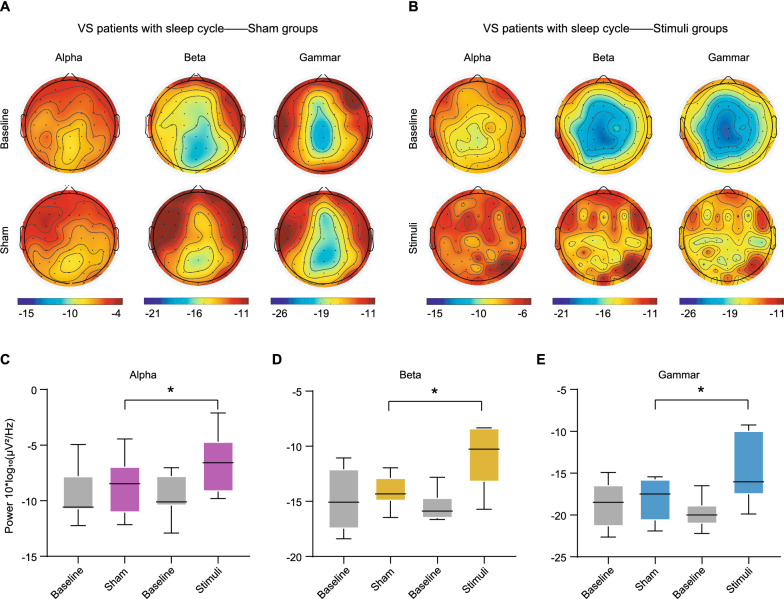
Changes in EEG power of VS patients with sleep cycles before and after treatments.** A** Topographical distribution of alpha, beta and gamma power on the scalp in the sham group. Red and blue indicate maximum and minimum EEG power (10*log_10_(μv^2^/Hz)), respectively. **B** Topographical distribution of alpha, beta and gamma power on the scalp in tDCS group. Red and blue indicate maximum and minimum EEG power (10*log_10_(μv^2^/Hz)) at each time point, respectively. **C**–**E** Comparison of EEG power in parietooccipital region before and after treatment both in sham group and tDCS group (Electrode number: 5,6,7,8,18,19,20,21,22,23,24,25,26,27,30,31,32,33,36,37,38,39,44,45,46,47,57), including alpha power (**C**), beta power (**D**) and gamma power (**E**). *P < 0.05, n.s: no significant difference; *P* values were determined using analysis of covariance (ANCOVA) and age was included as a covariate; tDCS: transcranial direct current stimulation; VS: vegetative state

To further investigate the role of the sleep cycle in the efficacy of tDCS in VS patients, a spectrum analysis of resting-state EEG was conducted before and after a 10-day tDCS treatment. For the VS patients with sleep cycles, increased alpha band, beta band and gamma band power were observed in topographic maps after tDCS, especially in the parieto-occipital regions (Fig. 3B), when compared to the sham group (Fig. 3A). For the VS patients without sleep cycles, there was no significant difference in the tDCS group (Additional file 3: Figure S2B) or the sham group (Additional file 3: Figure S2A). Furthermore, we extracted the relevant data of the parieto-occipital regions, revealing that VS patients with sleep cycles responded to tDCS better than the sham group, which was reflected by the increased power in α frequency bands (95%CI: 0.042–3.551, *P* = 0.005 Fig. 3C), β frequency bands (95%CI: 0.049–4.884, *P* = 0.046 Fig. 3D) and γ frequency bands (95%CI: 0.917–7.283, *P* = 0.048 Fig. 3E) respectively. However, these changes were not observed in the patients without sleep cycles (Additional file 3: Figure S2C–E).

**Fig. 4:**
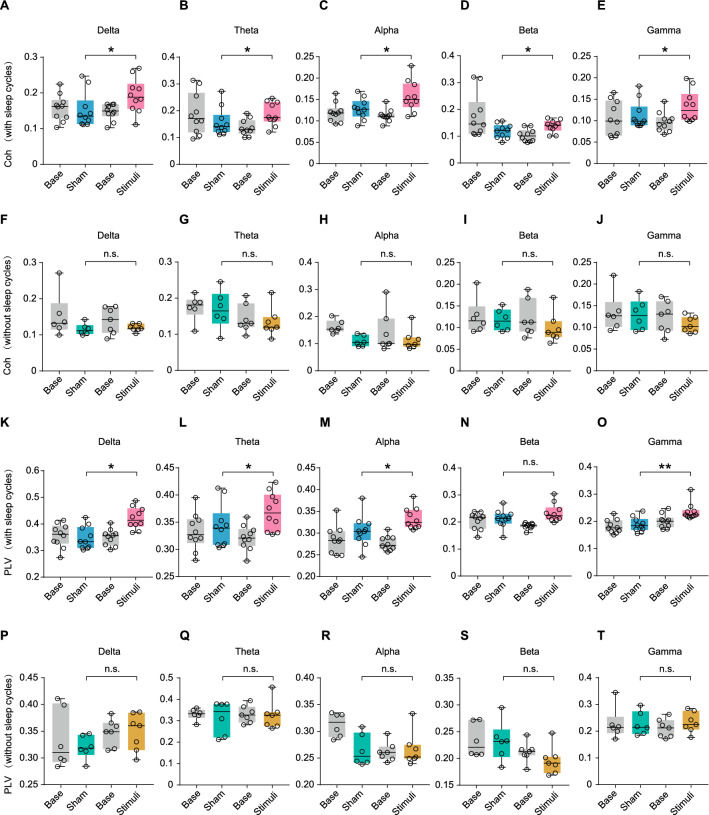
Changes in functional connectivity of parietooccipital region before and after treatments.** A**–**E** Comparison of coherence before and after treatment both in sham group and tDCS group of VS patients with sleep cycles, including delta (**A**), theta (**B**), alpha power (**C**), beta power (**D**) and gamma power (**E**). **F**–**J** Comparison of coherence before and after treatment both in sham group and tDCS group of VS patients without sleep cycles, including delta (**F**), theta (**G**), alpha power (**H**), beta power (**I**) and gamma power (**J**). **K**–**O** Comparison of PLV before and after treatment both in sham group and tDCS group of VS patients with sleep cycles, including delta (**K**), theta (**L**), alpha power (**M**), beta power (**N**) and gamma power (**O**). **P**–**T**) Comparison of PLV before and after treatment both in sham group and tDCS group of VS patients without sleep cycles, including delta (**P**), theta (**Q**), alpha power (**R**), beta power (**S**) and gamma power (**T**). Electrode number: 5,6,7,8,18,19,,20,21,22,23,24,25,26,27, 30,31,32,33,36,37,38,39,44,45,46,47,57. *P < 0.05, n.s: no significant difference; *P* values were determined using analysis of covariance (ANCOVA) and age was included as a covariate; Coh: coherence; PLV: phase locking value; tDCS: transcranial direct current stimulation; VS: vegetative state

To assess functional connectivity, network representations of the underlying neural dynamics are usually constructed to explore the similarities in the activity of different brain regions, which is widely applied to assess the level of consciousness [27, 28]. Therefore, we calculated the functional connectivity parameters of the relevant electrodes such as Coh and PLV. The Coh in all frequency bands (δ, θ, α, β, γ) was increased in VS patients with sleep cycles after a 10-day tDCS (Fig. 4A–E), but remained the same in those without sleep cycles (Fig. 4F–J). Furthermore, PLV showed the same trend in these groups (Fig. 4K–T), excluded the β band, suggesting that the efficacy of tDCS on post-stroke patients with prolonged DoC was associated with their sleep patterns.

**Fig. 5:**
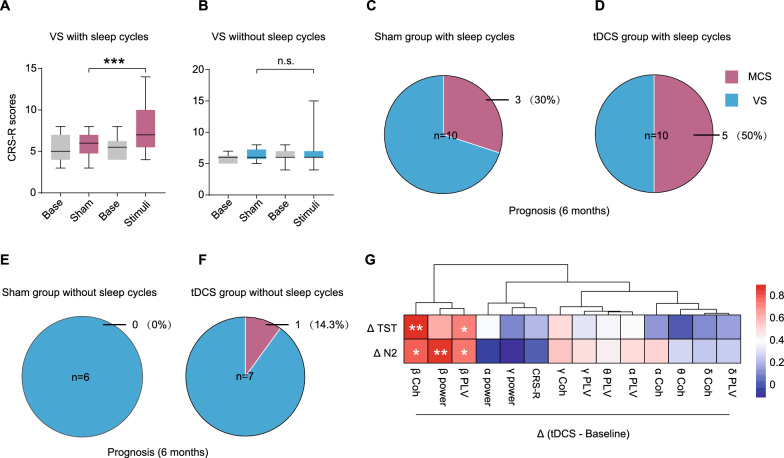
Changes in CRS-R scores and prognosis of VS patients with or without sleep cycles before and after treatments. Comparison of CRS-R scores before and after treatment in the sham group, including VS patients with sleep cycles (**A**) and VS patients without sleep cycles (**B**). Percentages of MCS patients who transformed from VS patients with sleep cycles in sham group (**C**) and tDCS group (**D**) after 6-month follow up. Percentages of MCS patients who transformed from VS patients without sleep cycles in the sham group (**E**) and tDCS group (**F**) after 6-month follow-up. **G **Heatmap of Pearson correlation analysis between altered TST, N2 stage and altered CRS-R scores, EEG power and functional connectivity. red means positive correlation, and blue means negative. *P < 0.05, **P < 0.01; ***P < 0.001, *P* values were determined using analysis of covariance (ANCOVA) and age was included as a covariate; Coh: coherence; TST: total sleep time; N2: nonrapid eye movement sleep 2 stage; CRS-R: Coma Recovery Scale-Revised scores; Coh: coherence; PLV: phase locking value; tDCS: transcranial direct current stimulation; MCS: minimally conscious state; VS: vegetative state

Moreover, the changes in CRS-R scores before and after the intervention were also explored. tDCS increased the CRS-R scores for the VS patients with sleep cycles (95%CI: 0.340–3.440, *P* < 0.001, Fig. 5A) but not for those without sleep cycles (Fig. 5B). Over the course of the 6-month follow-up (Additional file 2), 30% of the VS patients with sleep cycles in the sham group (Fig. 5C) and 50% in the tDCS group shifted into MCS (Fig. 5D). However, for the VS patients without sleep cycles, none of the patients in sham group and only one of the patients in tDCS group (14.3%) shifted into MCS (Fig. 5E, F). To investigate the correlation between sleep recovery and consciousness, correlation analysis was performed for the difference in CRS-R scores, EEG parameters and sleep parameters (Fig. 5G). Interestingly, the difference of TST and NREM stage 2 were significantly positively related to beta bands, including the beta power, Coh and PLV of beta bands, suggesting that the recovery of sleep may contribute to the improvement of brain function.

## Discussion

In our study, patients in MCS and VS who suffered from a stroke were selected and their sleep was monitored for 10 h at night. Patients with better consciousness had more NREM stage 2, which was associated with a better prognosis. However, the opposite was observed for SWS. Subsequently, the VS patients were divided into two subclasses according to their sleep patterns: with or without sleep cycles. The sleep patterns, EEG power, functional connectivity as well as CRS-R scores of VS patients with sleep cycles were improved after tDCS, but these improvements were not seen in patients without sleep cycles. Finally, altered TST and NREM stage 2 were significantly positively correlated with beta power, as well as Coh and PLV in beta bands. All the findings indicated that the sleep patterns, especially NREM stage 2, play an important role in the prognosis of prolonged DoC patients and influence the efficacy of tDCS. To our knowledge, this is the first study exploring the effects of sleep patterns in prolonged DoC patients caused by stroke, and the efficacy of tDCS in VS patients subclassified by sleep cycles. This study may provide new evidence for the importance of sleep patterns in assessing prolonged DoC and a new reference for the evaluation of the efficacy of tDCS.

The sleep patterns among patients with different levels of consciousness were widely studied, but it was difficult to draw reliable conclusions [29]. The main reasons include the different record parameters, relatively small cohort size and heterogeneity between patients, including etiology, various regions and severity of the damage, etc. [10]. In our study, the sleep patterns were only recorded at night (8 pm to 6 am), which matched the hospital schedule, because many passive rehabilitation exercises during the day and other environmental factors, such as light and sound, may influence the real sleep state of the patients. Moreover, to eliminate as many etiological biases as possible, we focused on post-stroke patients with prolonged DoC, whose prognoses were considered to be worse than those with traumatic brain injury [14].

Previous studies have shown that tDCS can not only regulate the duration and efficiency of sleep and improve the sleep quality [25, 30, 31], but it can also repair the abnormal brain network [32, 33]. In our results, for VS patients who suffered from a stroke, tDCS increased the TST and NREM stage 2 of those who have sleep cycles but had no effect in those without sleep cycles. It suggested that the plasticity of sleep in VS patients may depend on their original sleep cycles, and increased TST after unilateral tDCS may be driven by high sleep pressure from the activation of a ‘top-down’ pathway. In addition, The sleep spindles in NREM stage 2 generated during this stage are probably related to the strength and malleability of thalamocortical circuits [34], which are also regarded as the main neural mechanism in the maintenance of consciousness [35]. Increased NREM stage 2 suggested that tDCS may improve the connection of thalamus and cortex. Furthermore, for VS patients with sleep cycles, their EEG power and the functional connectivity of parieto-occipital regions as well as CRS-R scores have also improved accordingly. Compared to fronto-parietal regions, parieto-occipital regions are highly activated during some external goal-oriented tasks such as visual attention or functional cognitive memory tasks [36]. In our previous studies, the white matter connectivity and some brain subnetwork of parieto-occipital regions significantly decreased in VS patients compared to MCS patients [37, 38]. Therefore, polarity-specific changes in these regions, indexed by resting-state EEG power, Coh and PLV, are potential neural mechanisms of the effect of tDCS on post-stroke VS patients with sleep cycles. More importantly, patients with non-traumatic brain injury are generally considered to have a worse response, as well as a worse prognosis to tDCS than those with TBI [39]. In this study, post-stroke VS patients with sleep cycles showed significant improvement in both behavior and electrical activity, suggesting that evaluating the efficacy of tDCS based on a subclassified patient is likely to be necessary.

Another important highlight of this study is that the changes in the sleep stage, especially the NREM stage 2, have a significant positive correlation with changes in β power, Coh and PLV of β band. In general, increased β band is associated with a strengthening of sensory feedback in static motor control [40] and it can be induced by transcranial alternating current stimulation [41]. Therefore, it indicated that the recovery of the brain function was accompanied by an improvement in sleep in these patients. Notably, people have an increased NREM stage 2 and a decreased stage 1 and SWS, along with an increased β activity, when they are sedated with nitrazepam [42, 43]. In our study, patients who received any sedative were excluded. Therefore, the study highlights the possibility of a new mechanism of tDCS in prolonged DoC.

However, there are some limitations in our study. Firstly, the sample size of cohorts under the tDCS subclass study was relatively small and the results should be replicated in an independent study with a larger sample size in the future. Secondly, we did not consider the heterogeneity in the damage to different brain regions, even if the common etiology of these patients was a stroke. Thirdly, the stimulus target we selected was the dorsolateral prefrontal cortex, which has been the most widely explored. Investigation of individual stimulus sites based on different injured regions is necessary.

In summary, we found that NREM stage 2 and NREM stage 3 were associated with the severity and prognosis of post-stroke patients with prolonged DoC. Furthermore, tDCS can improve the sleep and brain function of VS patients who suffered from a stroke, and promote the recovery of their consciousness, only in those with sleep cycles. In these patients, changes in NREM stage 2 were synchronized with improved β bands in the brain.

## Supplementary Information

**Additional file 1.** The demographic and clinical characteristics of the all patients we detected.

**Additional file 2.** Scale scores and sleep cycles of patients enrolled in the intervention.

**Additional file 3: Figure S1. **Examples of sleep encephalogram for VS patients with or without sleep cycles. **A)** Example of sleep encephalogram for VS patients with sleep cycles. **B)** Example of sleep encephalogram for VS patients without sleep cycles. VS: vegetative state. **Figure S2. **Changes in EEG power of VS patients without sleep cycles before and after tDCS. **A)** Topographical distribution of alpha, beta and gamma power on the scalp in the sham group. Red and blue indicate maximum and minimum EEG power (10*log_10_(μv^2^/Hz)), respectively. **B)** Topographical distribution of alpha, beta and gamma power on the scalp in the tDCS group. Red and blue indicate maximum and minimum EEG power (10*log_10_(μv^2^/Hz)) at each time point, respectively. **C-E)** Comparison of EEG power in the parietooccipital region before and after treatment both in the sham group and the tDCS group (Electrode number: 5,6,7,8,18,19,,20,21,22,23,24,25,26,27, 30,31,32,33,36,37,38,39,44,45,46,47,57), including alpha power (C), beta power (D) and gamma power (E). n.s: no significant difference; *P* values were determined using analysis of covariance (ANCOVA) and age was included as a covariate; tDCS: transcranial direct current stimulation; VS: vegetative state.

## Data Availability

All data are included in the Source Data. The other data sets generated and/or analyzed in the current study are available from the corresponding authors upon reasonable request.
