# Supplementary material for: Sleep patterns correlates with the efficacy of tDCS on post-stroke patients with prolonged disorders of consciousness
Source: J Transl Med. 2022 Dec 15;20:601. doi: 10.1186/s12967-022-03710-2 (PMC9756665; doi:10.1186/s12967-022-03710-2)

Additional file 3: Figure S1

A

VS patients with sleep cycles

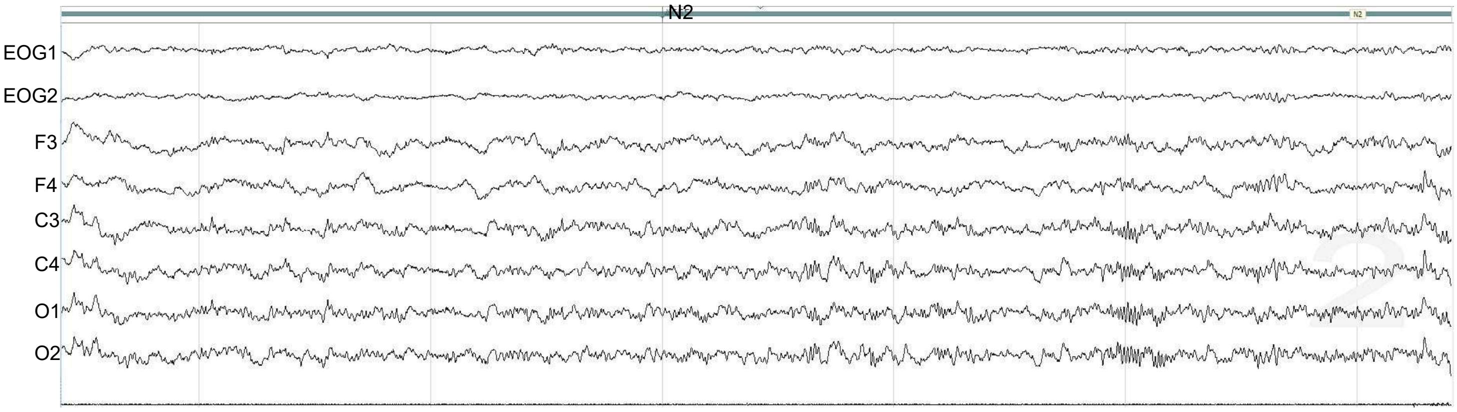

B

VS patients without sleep cycles

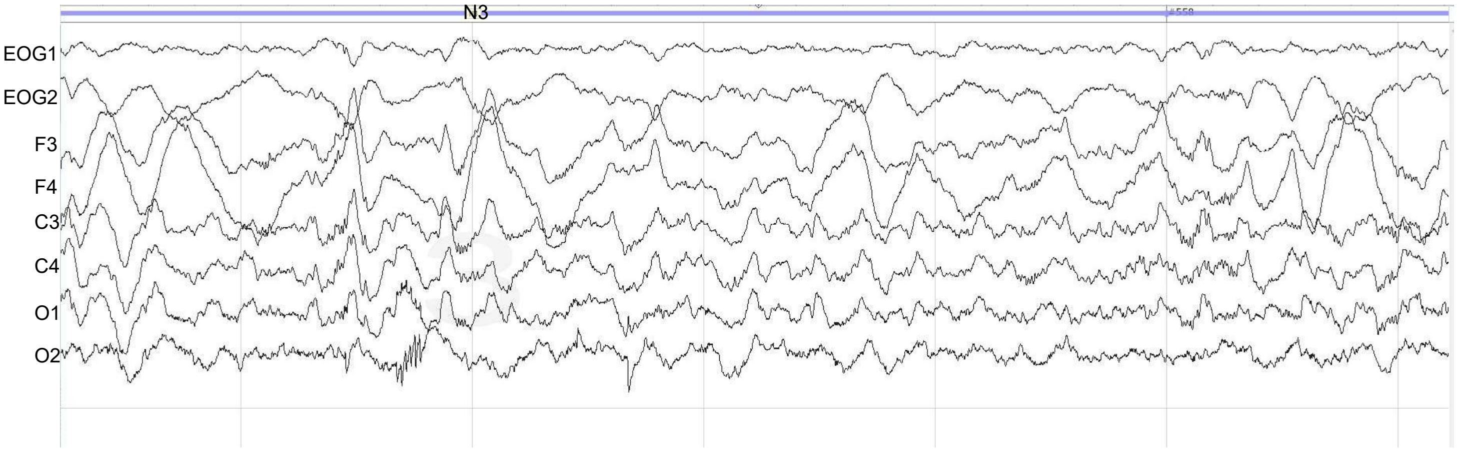

Additional file 3: Figure S

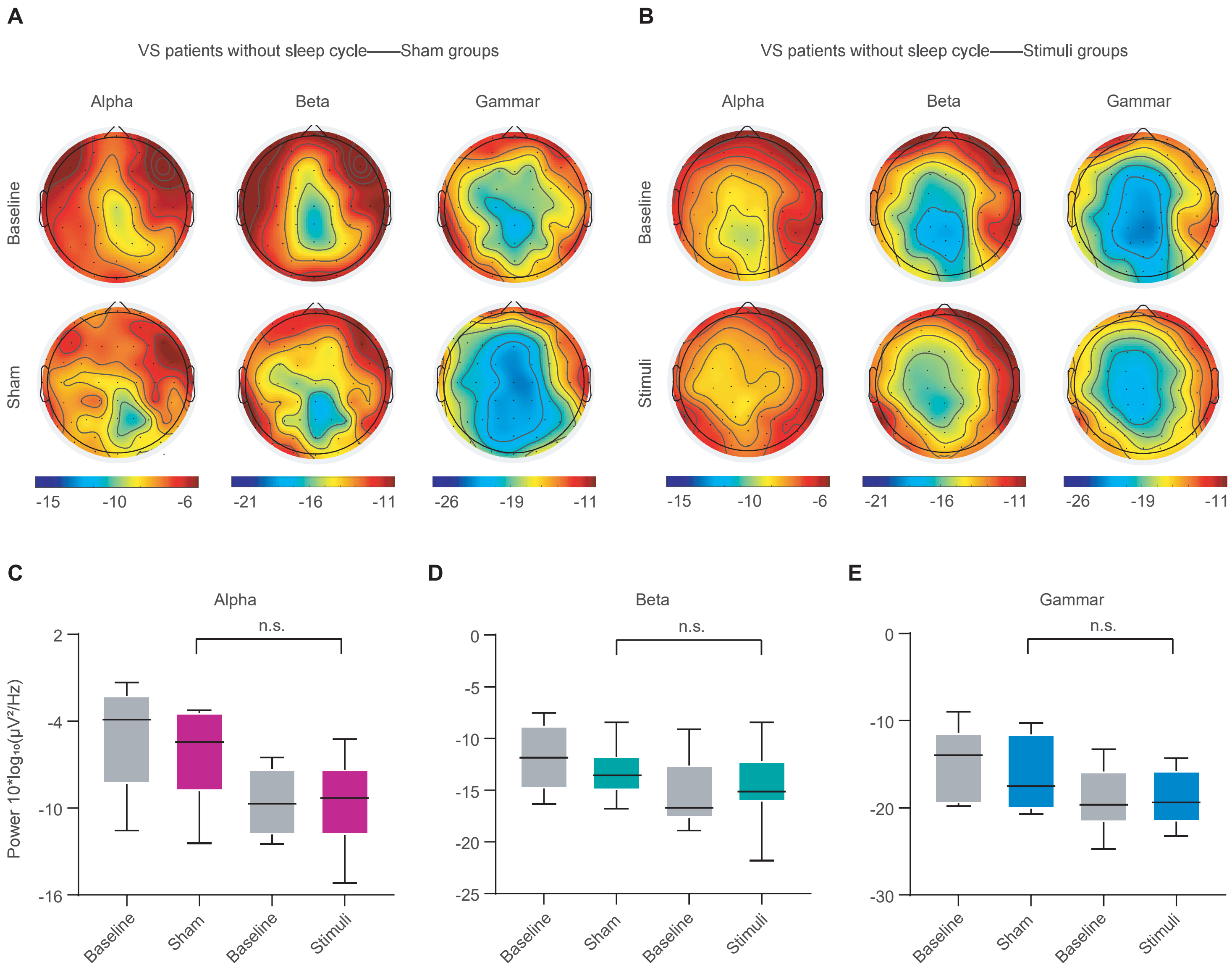

Supplement: Supplementary file 3 — Additional file 3: Figure S1. Examples of sleep encephalogram for VS patients with or without sleep cycles. A) Example of sleep encephalogram for VS patients with sleep cycles. B) Example of sleep encephalogram for VS patients without sleep cycles. VS: vegetative state. Figure S2. Changes in EEG power of VS patients without sleep cycles before and after tDCS. A) Topographical distribution of alpha, beta and gamma power on the scalp in the sham group. Red and blue indicate maximum and minimum EEG power (10*log10(μv2/Hz)), respectively. B) Topographical distribution of alpha, beta and gamma power on the scalp in the tDCS group. Red and blue indicate maximum and minimum EEG power (10*log10(μv2/Hz)) at each time point, respectively. C-E) Comparison of EEG power in the parietooccipital region before and after treatment both in the sham group and the tDCS group (Electrode number: 5,6,7,8,18,19,,20,21,22,23,24,25,26,27, 30,31,32,33,36,37,38,39,44,45,46,47,57), including alpha power (C), beta power (D) and gamma power (E). n.s: no significant difference; P values were determined using analysis of covariance (ANCOVA) and age was included as a covariate; tDCS: transcranial direct current stimulation; VS: vegetative state. [file 12967_2022_3710_MOESM3_ESM.pdf]
